# The epidemiology of Guillain-Barré Syndrome in U.S. military personnel: a case-control study

**DOI:** 10.1186/1756-0500-2-171

**Published:** 2009-08-26

**Authors:** Laura Nelson, Robert Gormley, Mark S Riddle, David R Tribble, Chad K Porter

**Affiliations:** 1 School of Public Health and Health Services, George Washington University, Washington, DC, USA; 2 Enteric Diseases Department, Naval Medical Research Center, Silver Spring, Maryland, USA; 3 Infectious Diseases Clinical Research Program, Uniformed Services University of the Health Sciences, Bethesda, Maryland, USA

## Abstract

**Background:**

Guillain-Barré Syndrome (GBS), the leading cause of acute flaccid paralysis worldwide, is an autoimmune disorder involving the loss of the myelin sheaths encasing peripheral nerve axons, leading to a loss of nerve signaling and typically ascending paralysis. A number of infectious triggers have been identified, with Campylobacter being most common. Limited data are available regarding GBS in U.S. service members at a high risk of exposure to numerous GBS-associated infectious agents.

**Findings:**

Medical encounter data were obtained from the Armed Forces Health Surveillance Center (Silver Spring, MD). Active duty personnel with an incident GBS diagnosis were matched by age, sex, and time with up to 4 controls. Demographic, antecedent infectious gastroenteritis (IGE), and deployment covariates were used to explore GBS risk in this population.

The overall incidence was 2.28/100,000 persons (95% confidence interval: 2.03–2.54) with 19.1% (60/314) receiving GBS-related medical care for more than one year. The majority of cases were male, Caucasian and under 25 years of age. There was an increased risk of GBS three months following a documented episode of IGE (Odds Ratio: 5.33; p = 0.03). We also found an association with service in the Air Force and Navy (compared to Army personnel) with odds ratios of 1.39 (p = 0.05) and 1.44 (p = 0.02), respectively.

**Conclusion:**

GBS incidence in the U.S. military is slightly higher than the general population and is associated with an antecedent IGE. Future studies are warranted to assess whether there are GBS-associated infectious or environmental exposures inherent to military populations.

## Background

Guillain-Barré Syndrome (GBS) is the leading cause of acute flaccid paralysis with an estimated worldwide incidence of 1.1–1.8/100,000 persons and has been associated with $1.7 billion in yearly costs in the United States alone [1,2]. Cases range from mild to severe disease and recovery depends on disease severity. Symptoms usually take 6 to 18 months to fully resolve, although a small proportion may require prolonged hospitalization or care [3-5].

The association between GBS and antecedent infection has been well described, with *Campylobacter jejuni*, Cytomegalovirus (CMV), and Epstein-Barr virus (EBV) commonly identified and C. jejuni being by far the most frequent [6-11]. Interestingly, *C. jejuni*-associated GBS may be associated with a more severe clinical presentation [12].

Campylobacter is a leading bacterial cause of IGE world-wide [13]. A number of studies also document *C. jejuni *as a leading cause of travelers' diarrhea (TD) in U.S. military personnel, particularly in Southeast Asia [14-16]. Additionally, military personnel are exposed to numerous deployment-related vaccinations [17], which have also been linked to several autoimmune diseases including GBS [18,19].

Little data on GBS epidemiology in military populations are available. Using data from the Defense Medical Surveillance System (DMSS), from 1998 to 2007, we identified incident cases of GBS in active duty military personnel and, utilizing a match case-control study design, evaluated the association between GBS and acute gastrointestinal infection and deployment.

## Methods

Data were obtained from the Armed Forces Health Surveillance Center (AFHSC) which oversees the Defense Medical Surveillance System (DMSS), a database containing all medical encounters at military treatment facilities for all active duty US military from 1998 to the present [20]. Medical information was linked to demographic and deployment databases. Prior to provision of the final dataset for analysis, study identification numbers were assigned and identifiable information was removed.

Cases were active duty US military personnel with a GBS diagnosis (ICD9-CM code 357.0) between 1999 and 2007. We required two separate GBS-associated medical encounters within 1 year of initial diagnosis to increase specificity. Controls (≥4 per case) were identified from the same medical encounter dataset, and were matched on age (within 1 year), sex, and time (within 1 year) of an unrelated medical encounter.

Additional covariates evaluated included type of medical visit (inpatient or outpatient), marital status, rank, service type, education and race. An antecedent episode of IGE in the 1 year prior to censure was evaluated as a risk factor for GBS. Deployment to a region of high TD risk (including Iraq, Afghanistan, Southwest Asia and the Persian Gulf) in the year preceding censure was also evaluated as a surrogate for prior IGE.

For each case, all GBS-related medical encounters were evaluated. Cases with a year or more of GBS-related encounters were classified as "chronic". The incidence of GBS (with exact 95% confidence intervals) was estimated using the number of cases and the number of active duty US military personnel (from the Defense Medical Epidemiology Database). Poisson regression was used to assess temporal changes in incidence. The association between GBS, antecedent IGE, operational deployment and other covariates were explored using univariate conditional logistic regression methods. Odds ratios (OR) were calculated with Wald 95% confidence intervals. All data were analyzed using SAS v. 9.1 (SAS Institute, Cary, NC) with an *alpha *of 0.05.

## Results

The majority of cases were male (n = 262; 83.4%), between the ages of 20 and 24 (n = 101; 32.2%), Caucasian (n = 210; 66.9%) and single (n = 153; 48.7%) (table 1). Cases and controls were mostly enlisted (83.4% and 85.1%, respectively). The majority of cases (n = 226; 72.0%) and controls (n = 908; 72.3%) had no more than a high school education. Cases were more commonly Navy personnel (n = 101; 32.2%) than other branches of service while controls were more commonly in the Army (n = 466; 37.1%). Controls had no outcomes associated with enteric infections. The most common ICD-9 code for controls was V70.5, a routine health exam, accounting for 10.9% of included controls. No other single ICD-9 accounted for more than 5% of the controls.

**Table 1:** Demographic characteristics of U.S. military service members diagnosed with Guillain-Barré Syndrome between 1999 and 2007 and their matched controls

	**Cases**	**Controls**
N	314	1,256

Age^1^	28.7 (9.3)	28.6 (9.0)

Sex^2^		

Female	52 (16.6)	208 (16.6)

Male	262 (83. 4)	1048 (83.4)

Race^2^		

Black	55 (17.5)	215 (17.1)

White	210 (66.9)	794 (63.2)

Other	49 (15.6)	247 (19. 7)

Marital status^2,3^		

Married	148 (47.1)	555 (44.2)

Single	153 (48.7)	651 (51.8)

Other	9 (2.9)	47 (3.7)

Rank^2^		

Enlisted	262 (83.4)	1069 (85.1)

Officer	47 (15.0)	177 (14.1)

Warrant Officer	5 (1.6)	10 (0.8)

Service type^2^		

Army	93 (29.6)	466 (37.1)

Air Force	80 (25.5)	289 (23.0)

Marines	40 (12.7)	147 (11.7)

Navy	101 (32.2)	354 (28.2)

Education level^2,4^		

High school (or equivalent)	226 (72.0)	908 (72.3)

Some college	27 (8.6)	95 (7.6)

Bachelor's degree	27 (8.6)	132 (10.5)

Master's degree	14 (4.5)	65 (5.2)

Doctorate degree	9 (2.9)	16 (1.3)

^1 ^Mean (standard deviation)

^2 ^Number (percent)

^3 ^4 cases (1.3%) and 3 controls (0.2%) missing data

^4 ^11 cases (3.5%) and 40 controls (3.2%) missing data

**Figure 1:**
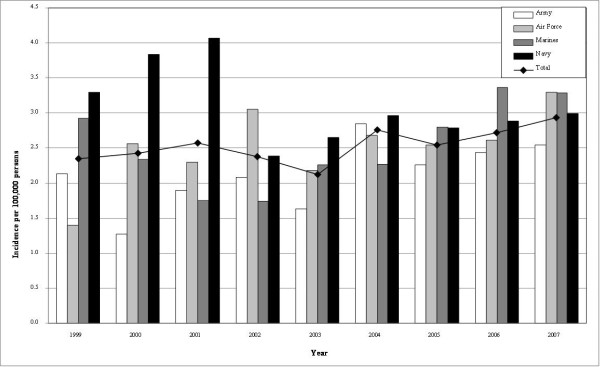
**Incidence of GBS cases stratified by branch of military service**.

Overall, GBS incidence was 2.28 (95% C.I.: 2.03, 2.54) per 100,000 person-years (table 2). The incidence increased from 2.35 in 1999 to 2.94 in 2007 with a spike of cases in 2004 (figure 1). Although no significant changes in GBS incidence were observed over the period of observation, general fluctuations were seen. Navy personnel had the highest incidence of GBS in 5 of 9 (figure 1). Additionally, non-white and non-black females had the highest incidence in five out of nine years with no observable trends. The youngest age category (< 20 years of age) had an increasing GBS incidence from 1999 to 2005 and had the highest incidence during 4 of those years. Persons ≥ 40 years of age had a similar increasing trend from 2003 to 2007.

**Table 2:** Incidence of GBS and number of GBS-related medical encounters in U.S. military service members diagnosed between 1999 and 2007 stratified by demographic covariates

**Covariate**	**Incidence**^**1**^	**Number of GBS-related medical visits**^**2**^	**Number of GBS-related medical visits 1 month post-censure**^**2**^	**Number of GBS-related medical visits 1 year post-censure**^**2**^	**Number of cases receiving GBS-related medical care beyond 1 year post-censure**^**3**^
Female	2.59 (1.93, 3.39)	6 (3, 18)	4 (2, 8)	6 (3, 16.5)	13 (25%)

Male	2.22 (1.96, 2.51)	9 (4, 17)	4 (2, 9)	8 (3, 17)	47 (18%)

< 20	3.48 (2.47, 4.75)	12 (7, 21)	7 (5. 12)	12 (7, 21)	4 (10%)

20–24	2.23 (1.82, 2.71)	10 (4, 18)	4 (2, 10)	10 (4, 18)	15 (15%)

25–29	2.21 (1.70, 2.83)	6 (3, 17)	4 (2, 9)	6 (3, 16)	9 (14%)

30–34	1.61 (1.11, 2.26)	5 (2, 15)	3 (2, 7)	5 (2, 15)	7 (21%)

35–39	1.73 (1.19, 2.45)	8.5 (3.5, 13.5)	2 (2, 5.5)	7.5 (3, 12.5)	10 (31%)

>= 40	3.29 (2.41, 4.39)	6.5 (3, 22)	3 (1, 9)	5.5 (2, 20)	15 (33%)

Black	2.07 (1.56, 2.70)	8 (3, 17)	5 (2, 13)	8 (3, 17)	8 (14%)

White	2.22 (1.93, 2.54)	8 (4, 17)	4 (2, 9)	8 (3, 17)	45 (21%)

Other	2.98 (2.21, 3.92)	10 (3, 18)	4 (2, 10)	10 (3, 18)	7 (14%)

Total	2.28 (2.03, 2.54)	8.5 (3, 17)	4 (2, 9)	8 (3, 17)	60 (19.1%)

^1 ^Incidence per 100,000 with 95% exact binomial confidence intervals

^2 ^Presented as a median number of visits with interquartile ranges

^3 ^Presented number of cases with percent of subgroup

The censoring diagnosis for most cases (52.5%) occurred in an outpatient setting although 225 (71.7%) had an inpatient GBS-related medical encounter during the course of their medical care. After initial case presentation, common GBS-related follow-up medical encounters included: physical therapy (n = 53; 16.9%), occupational therapy (n = 24; 7.6%), rehabilitation (n = 49; 15.6%) and speech therapy (5; 1.6%).

The median number of GBS-related medical visits is shown in table 2. Although there was a trend toward a higher number of follow-up visits for males, younger cases (those in the 20 year age group), and those of race "other," these differences were not significant. A total of 19.1% of cases received GBS-related medical care beyond 1 year after initial presentation.

In the three months prior to censure, cases had a higher odds of a documented antecedent IGE compared to matched controls (OR: 5.33; 95% C.I.: 1.19, 23.82), although no specific IGE etiology was found (Table 3). We were unable to identify a significant association between GBS and prior deployment to a region with high TD risk in the year preceding censure. However, 11 cases (3.5%) had their initial GBS encounter during a deployment to one of those regions. Compared to their Army counterparts, Air Force (OR: 1.4; 95% C.I.: 1.0, 1.9) and Navy (OR: 1.4; 95% CI: 1.1, 2.0) personnel had a higher odds of GBS. Education also appeared to be associated with an increased odds of GBS with a higher odds in those with doctorate degrees compared to those with no greater than a high school education (OR: 2.28; 95% CI: 1.00 to 5.23). This association was not observed across other education categories.

**Table 3:** Risk factors and covariates associated with GBS among U.S. military personnel diagnosed between 1999 and 2007 stratified by demographic covariates

**Covariate**	**OR (95% CI)**
**Race**	

Non-white^1^	1.00

White	1.17 (0.90, 1.52)

**Rank**	

Enlisted^1^	1.00

Officer/Warrant Officer	1.17 (0.80, 1.69)

**Marital status**	

Not married^1^	1.00

Married	1.22 (0.89, 1.68)

**Service Type**	

Army^1^	1.00

Air Force	1.39 (1.00, 1.95)

Navy	1.43 (1.05, 1.97)

Marines	1.38 (0.90, 2.12)

**Education**	

High school (or equivalent)^1^	1.00

Some college	1.14 (0.72, 1.81)

Bachelor's degree	0.83 (0.53, 1.32)

Master's degree	0.87 (0.45, 1.68)

Doctorial degree	2.41 (0.97, 5.95)

**IGE 3 months pre-censure**	5.33 (1.19, 23.82)

**Deployment 3 months pre-censure**^**2**^	0.96 (0.56, 1.64)

^1 ^Reference category

^2 ^Deployment to Iraq, Afghanistan, Southwest Asia and/or Persian Gulf evaluated

## Discussion

We found a slightly higher incidence of GBS (2.3/100,000) in an active duty U.S. military population compared to the general population estimates of 1.1 to 1.8/100,000 recently reported [2]. This may represent a true GBS risk among U.S. military personnel due to an increased risk of exposures to known infectious triggers, unknown triggers (infectious or non-infectious), or an artifact of the methodology utilized. McGrogan et al reported that "retrospective database studies" found an overall higher GBS incidence compared to prospective studies or retrospective medical record reviews [2]. This may reflect incomplete or incorrect medical coding in database studies, inadequate case-finding procedures or poorly defined estimates of at risk populations in other study designs. In an effort to minimize coding errors, we required a GBS diagnosis on at least two separate medical encounters, eliminating 285 possible cases and effectively halving our incidence estimate. Of these 285, a majority (n = 207) had an ICD9-CM of 357.0 in the first diagnostic position and 33 had their lone GBS-related medical encounter at an inpatient facility. Thus, our methodology may have also biased our incidence estimates downward.

We found a bimodal distribution of incidence with highest rates in those under 25 and over 40 years of age. Although the biological mechanisms are uncertain and the epidemiologic data are inconsistent, others have reported similar findings. One hypothesis is that the peak in late adolescence and early adulthood is associated with a secondary peak in *C. jejuni *incidence in that age group (in developed world populations), whereas the peak in an elderly population may be associated with failed immunosuppression mechanisms [21]. Others have hypothesized an age-specific differential risk of *Campylobacter*-attributable GBS [10]. Further research is needed to fully understand this effect.

Prior IGE was associated with an increased GBS risk. Although we were unable to attribute this increased risk with a specific organism, the known Campylobacter association is the most likely causative agent [11]. Unfortunately, the utilization of ICD9-CM codes to identify infectious exposures, specifically those of GI origin, is limited by the scarcity of microbiologic work-up completed on reporting patients. This has been well-described and continues to be a source of likely non-differential exposure misclassification in these types of studies [22]. Future research should consider a more extensive case history to identify common triggers within the active duty population.

We found a significant increased odds of GBS among Air Force and Navy personnel (compared to Army), and among those with a doctorate degree (compared to no more than a high school education). This may reflect a subgroup that was older than the overall study population (mean: 42.9; standard deviation: 9.5) and may disproportionately represent the ≥ 40 age group, known to have higher GBS rates [21]. Due to age-matching, we were unable to assess age as an independent risk-factor. The association with branch of service has not been previously described. Possible contributing factors could be differences in infectious exposures unique to these 2 branches and further study on the exact etiology of infectious triggers in this population is needed. Additionally, these findings may be associated with differential healthcare utilization in these subgroups. While there is open access to care for all active duty personnel, there may be differential health-seeking behaviors.

GBS is known to cause chronic disability in a minor proportion if cases [9,21]. In our study, approximately 20% of cases had multiple medical visits 1 year post-diagnosis. This may be influenced by the fact that active duty military personnel have essentially unfettered access to free medical care. However, the proportion of "chronic" cases was similar to that reported by Forsberg et al albeit in an older Swedish population [23]. In evaluating the diagnostic codes of follow-on GBS-related medical visits, the most common were associated with physical therapy, rehabilitation, and speech therapy. These data underscore the fact that while GBS sequelae may be rare, the morbidity in terms of loss of function and disability need to be explored and considered when evaluating the importance of infectious disease prevention.

We did not evaluate the differential odds of exposure to other infectious agents with known GBS associations (e.g., CMV, EBV), nor did we evaluate the association with antecedent vaccination. We did, however, evaluate deployment to military operations in the Middle East and Southwest Asia in the year preceding censure as a potential surrogate for military relevant exposures and found no association with GBS. We were unable to assess peacetime deployments, specifically those to Southeast Asia, an area with high rates of Campylobacter [24-26]. This represents a significant limitation in the utilization of deployment as a surrogate for potentially relevant exposures.

As mentioned, utilizing a medical encounter database has inherent limitations and introduces some biases. First, data coding errors and misclassification are inherent and such errors are the source of numerous peer-reviewed publications [27,28]. Another factor we were unable to assess was infectious exposures occurring during deployment. The DMSS only includes medical encounters at U.S.-based medical treatment facilities. This represents a decreased likelihood of capturing potentially important exposures, including IGE, which occur during deployment limiting the generalizability of these findings. Case-finding and/or seroepidemiologic studies may more accurately identify prior exposures and outcomes and minimize the biases inherent in utilizing medical encounter data.

In summary, this preliminary study found IGE as a trigger for GBS, however questions remain regarding the etiology of such infections, and further study is needed to identify other common and potentially preventable triggers in this population. Regardless, we have described a potentially important infectious sequelae which may not factor in to decreased mission readiness in a deployed operational setting, but may present a significant preventable burden from an individual's and the military health and disability system's perspective.

## Competing interests

The authors declare that they have no competing interests.

## Authors' contributions

MR, CP, DT and RG conceived of the study, and participated in its design and coordination. LN and CP performed data analysis and interpretation and drafted the manuscript. MR and DT participated in data interpretation and manuscript writing. All authors read and approved the final manuscript.
